# Synovial sarcoma of the dorsal aspect of the hand: a case report

**DOI:** 10.1186/s13256-024-04469-4

**Published:** 2024-03-15

**Authors:** Farhad Bizhanzadeh, Fateme Heydari, Ronak Rashedi, Mohammadhosein Ramezanirad, Amir Reza Bahadori

**Affiliations:** 1 Shiraz University of Medical Sciences, Shiraz, Fars Iran; 2 School of Medicine, ShahidBeheshti University of Medical Sciences, Tehran, Iran; 3 Student Research Committee, School of Medicine, ShahidBeheshti University of Medical Sciences, Tehran, Iran

**Keywords:** Synovial sarcoma, Hand, Recurrence, Case report

## Abstract

**Background:**

Synovial sarcoma is a rare soft tissue sarcoma, with incidences of 0.81/1,000,000 in children and 1.42/1,000,000 in adults. It is most commonly found in soft tissue and rarely in bone. It often has a slow growth pattern and a benign radiologic appearance.

**Case presentation:**

This study reports a case of metacarpal synovial sarcoma occurring in the hand-wrist of a 32-year-old Iranian man presented with the chief complaint of a lump on the dorsal ulnar side of his left hand and wrist. Initially, the first physician suspected the case to be a ganglion cyst. After two months of conservative treatment, the size of the lesion gradually increased. Magnetic resonance imaging (MRI) was performed and after an excisional biopsy and a postoperative histological analysis, the tumor was identified as a synovial sarcoma. The patient underwent a scheduled surgical procedure. Unfortunately, he had poor follow-ups and brought the pathologic results two months later when, the tumor had incredible growth, which makes this presentation rare.

**Conclusions:**

Since early diagnosis can lead to higher survival rates, this report increases doctors' awareness of this extremely malignant tumor that is rarely seen.

## Background

Synovial sarcoma is a comparatively infrequent malignancy characterized by a soft tissue sarcoma of ambiguous differentiation. Five to 10% of all soft tissue sarcomas are synovial sarcomas [1]. Each year, approximately 1000 persons in the United States are diagnosed with synovial sarcoma, with age-adjusted incidences of 0.81/1,000,000 in children and 1.42/1,000,000 in adults [2].

We can find this neoplasm almost anywhere in the body. It usually affects deep soft tissue in the lower extremities. However, it can also affect the head and neck [3].

Synovial sarcoma is most commonly found in soft tissue and rarely in bone. However, few studies on intraosseous synovial sarcoma have been conducted [4, 5], and reports on cases with cytogenetic or molecular approval are even more infrequent [6–9].

According to reports, it can also occur in the central nervous system, peripheral nerves, oral cavity, retro-peritoneum, peritoneum, viscera (pleura, heart, kidney, lung), and mediastinum. It often has a slow growth pattern and a benign radiologic appearance. Clinically, synovial sarcomas can cause pain depending on where they are in relation to the nerves. A few authors have reported that synovial sarcomas are more likely than other sarcomas to cause persistent pain at the site of the tumor before swelling appears [10].

This paper describes a case of metacarpal synovial sarcoma. The tumor diagnosed as synovial sarcoma after a postoperative histopathological examination following an excisional biopsy. A planned surgery performed on the patient. However, in the two months that we were waiting for pathologic experiment answers, the tumor had incredible growth, which makes this presentation rare.

## Case presentation

A 32-year-old Iranian man with an unremarkable medical history presented with the chief complaint of a lump on the dorsal ulnar side of his right hand and wrist. He first noticed his symptoms approximately one month earlier. The patient reported that the mass had been there for one month and had been growing steadily over the previous month. There was no prior trauma history or constitutional symptoms, and his past medical, and family history was otherwise unremarkable. He noted some swelling in the left dorsal area but no fever, chills, or pain. Furthermore, in part of his social history, he did not abuse alcohol, drugs, or cigarettes. Also, at the time of admission, his vital signs were in normal range (pulse rate = 75, blood pressure = 118/83, temperature = 36.7, O2 saturation = 98%). In addition, as Table 1 depict all patient’s laboratory findings except 25-Hydroxy Vitamin D3 were normal. Thus, for his low level of 25-Hydroxy Vitamin D3, supplement medication (a tablet of Vitamin D3 1000 mg daily) was started.

**Table 1:** Patient’s laboratory test findings at the time of admission

Laboratory test	Result	Unit	Reference range
*CBC*			
WBC	6.22	10^3^ /µL	4.5–11.5
RBC	4.8	10^6^/µL	4–5.4
Hemoglobin	13.8	g/dL	12–15
Hematocrit	41	%	35–49
M.C.V	86.4	fL	80–99
M.C.H	29.9	pg	26–32
M.C.H.C	33.6	g/dL	32–36
Platelets	222	10^3^/µL	150–450
RDW-CV	14	%	11–14.5
*Coagulation test*			
PT (Patient)	14.0	Sec	14.0–16.5
INR	1.0		
PT (Control)	14.0	Sec	14.0
PTT	33	Sec	30–40
*Biochemistry*			
BUN	16.5	mg/dL	7–18.6
Creatinine	0.63	mg/dL	0.5–1.4
SGOT	21	IU/L	Up to 40
SGPT	19	IU/L	Up to 40
Alkaline phosphatase	210	U/L	64–306
Protein total	8.6	g/dL	6.6–8.8
Albumin	5.0	g/dL	3.5–5.2
25-Hydroxy Vitamin D3 (Low)	10.0	ng/ml	Deficient: < 20
*Serology*			
CRP (Quantitative)	1.9	mg/L	Up to 6
Hematology			
ESR 1 hour	4	mm/h	Up to 20

*CBC* Complete Blood Count, *WBC* Wight Blood Cell, *RBC* Red Blood Cell, *M.C.V.* Mea Corpuscular Volume, *M.C.H.* Mean Corpuscular Hemoglobin, *M.C.H.C* Mean Corpuscular Hemoglobin Concentration, *RDW-CV* Red Cell Distribution Width–Coefficient of Variation, *PT* Prothrombin Time, *INR* International Normalized Ratio, *PTT* Partial Thromboplastin Time, *BUN* Blood Urea Nitrogen, *SGOT* Serum Glutamic Oxaloacetic Transaminase, *SGPT* Serum Glutamic Pyruvic Transaminase, *CRP* C-Reactive Protein, *ESR* Erythrocyte Sedimentation Rate

Standard hand-wrist radiographs were taken to rule out any foreign body or fracture.

**Fig. 1:**
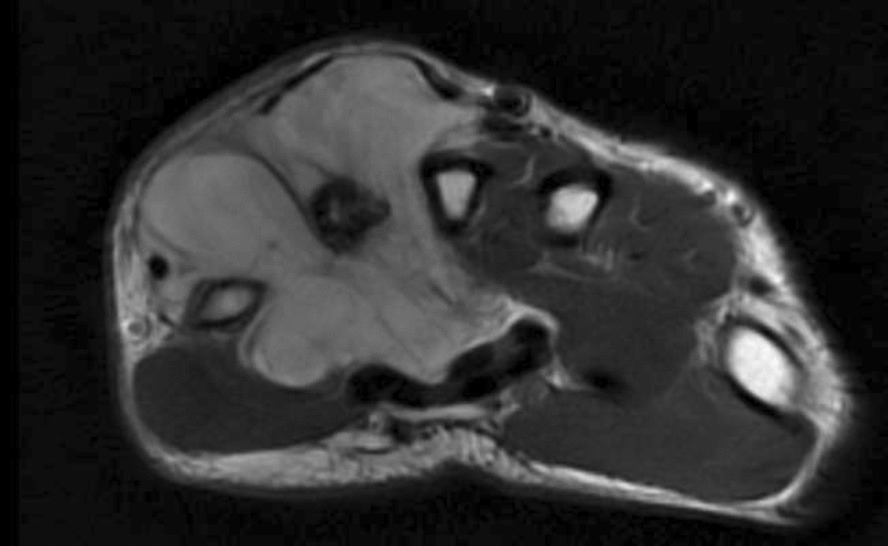
Magnetic resonance image of the right hand showing a heterogeneous mass-like t2 signal intensity lesion encasing the second metacarpal with pressure effect over flexor and extensor tendons

**Fig. 2:**
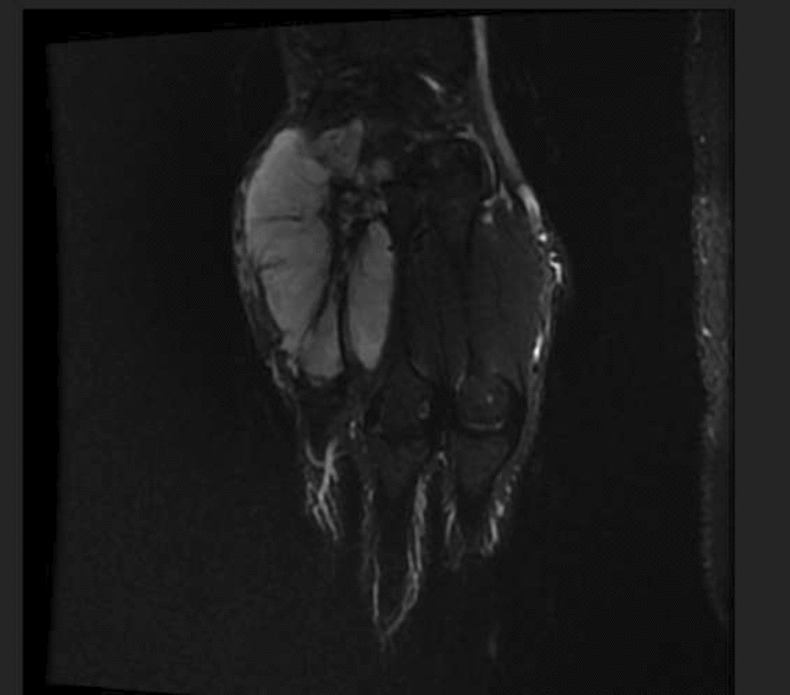
Magnetic resonance image of the right hand showing a heterogeneous mass-like t2 signal intensity lesion encasing the second metacarpal with pressure effect over flexor and extensor tendons

There was no evidence of bone involvement, and no foreign bodies or breaks in any cortical margins were noted. The primary physician clinically suspected the case as a ganglion cyst during that period. After receiving conservative treatment (a tablet of Naproxen 500 mg when he suffers from pain) for 2 months, the size of the lesion gradually increased. On clinical examination, a mass of size 30 × 20 mm was found between the second and third metacarpal bones of the dorsal surface of the left hand, extending up to the proximal of the metacarpal bones and the distal part of the wrist joint. Moreover, in terms of neurovascular examination patient did not have any significant abnormalities, despite the tumor size. The patient could flex and extend his wrist suitably. His deep tendon reflex was normal. Moreover, his sensation and precipitation were literally acceptable. As well, his muscle power was 5 out of 5. In addition, Magnetic Resonance Imaging (MRI) was performed, and the image was reviewed by a radiologist. The image revealed a hypersignal lesion in T2 that was iso to a hypersignal lesion in T1 in the deep soft tissue of the middle and medial side of the hand in the 3rd and 4th web with encasement of the 4th metacarpal bone. The lesion was suggestive of a cystic structure with fine internal septation and heterogeneous signal intensity. A deviation of the flexor tendon was observed due to the mass effect of this lesion. Additionally, displacement of the extensor tendon of the hand was observed in the 3rd, 4th, and 5th fingers (Figs. 1, 2).

With the patient under local anesthesia, an excisional biopsy was performed, which revealed a gray gelatinous, loose mass adhering to the lumbrical and interosseous muscles and invading the carpometacarpal joint. The mass was excised in total and sent to the pathology laboratory for review.

**Fig. 3:**
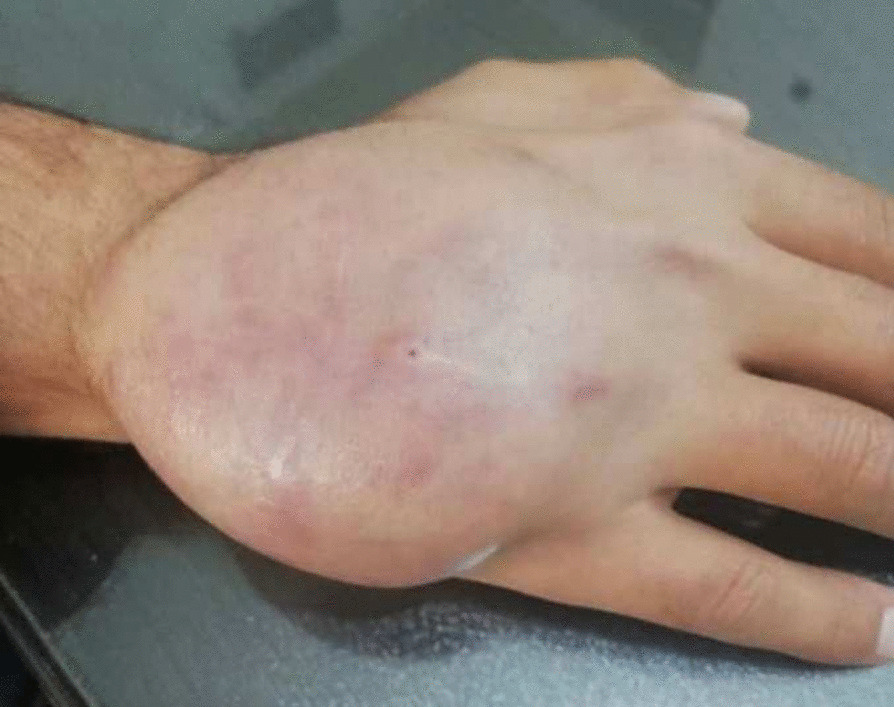
Sarcoma recurrence was discovered on the dorsal surface of the left hand two months after excisional biopsy

Unfortunately, the patient had poor follow-up and did not return to the clinic after 2 months when he reported a recurrent lesion at the same location (Fig. 3). Furthermore, in that follow-up, he brought the pathology report which revealed synovial sarcoma (biphasic). Considering the pathology report, abdominal—pelvic, and thoracic CT scans were requested for him and the result of the aforementioned images was literally normal. The patient was referred to an orthopedic oncologist. As a part of patient treatment, amputation was also suggested to the patient However, the patient refused to start his treatment and had no more follow-ups.

## Discussion

The current case report illustrated the metacarpal synovial sarcoma, which presented in the left hand-wrist of a 32-year-old Iranian man who consult a physician with the chief complaint of a lump on the dorsal ulnar side of his left hand and wrist. Therefore, MRI was applied for him and after an excisional biopsy, the tumor was identified as a synovial sarcoma.

Synovial sarcoma is a relatively rare but distinctly malignant type of soft tissue sarcoma, making up approximately 5–10% of all soft tissue sarcomas. Furthermore, it is more infrequent for a synovial sarcoma to affect the hand-wrist anatomical region, accounting for only 4–8.5% of all synovial sarcomas. Consequently, this malignant tumor is highly vulnerable to misdiagnosis due to its low suspicion rate by physicians and pathologists [11, 12].

Herein, we have reported a case of synovial sarcoma in a 32-year-old male involving the dorsal surface of the hand. Synovial sarcoma of the hand-wrist anatomical region mostly tends to affect the palmar surface of the hand, and involvement of the dorsal surface is less frequently reported [11].

The reported patient was first misdiagnosed and thus mistreated as a benign case of ganglion cyst. Misdiagnosis of synovial sarcoma has been frequently reported, not only because physicians do not encounter this tumor commonly in their everyday practice but also because it is known for masquerading the symptoms of other disorders. Karki *et al.* reported a case of synovial sarcoma of the palm misdiagnosed as a neural tumor [13].

The patient in this report came back with a relapse of this tumor after two months, showing a fast growth pattern. Relapse of synovial sarcoma is infrequently seen, accounting for only 25–32% of all patients. Nevertheless, the prognosis after relapse is relatively poor, and only half of patients can be cured by successive treatments [14, 15]. Being older at the time of presentation, male gender, a larger tumor size, bone or neurovascular proliferation of the tumor, central location, and incomplete surgical excision are known risk factors for the recurrence of synovial sarcoma [11].

Synovial sarcoma of the hand is known to have a slow growth pattern [12, 14]. Contrary to the previously reported cases of synovial sarcoma of the hand, our patient showed a unique and rapid growth pattern, reaching a tremendously large size only in two months.

## Conclusion

This report aims to draw the attention of physicians to this rarely-seen yet tremendously malignant tumor, as a high suspicion is key to early diagnosis, and early diagnosis can result in increased survival rates.

## Data Availability

If supporting data is needed contact the correspondence author.

## Abbreviation

MRI: Magnetic resonance imaging
